# Epidemiology of Sleeping Sickness in Boffa (Guinea): Where Are the Trypanosomes?

**DOI:** 10.1371/journal.pntd.0001949

**Published:** 2012-12-13

**Authors:** Moise Saa Kagbadouno, Mamadou Camara, Jeremi Rouamba, Jean-Baptiste Rayaisse, Ibrahima Sory Traoré, Oumou Camara, Mory Fassou Onikoyamou, Fabrice Courtin, Sophie Ravel, Thierry de Meeûs, Bruno Bucheton, Vincent Jamonneau, Philippe Solano

**Affiliations:** 1 Programme National de Lutte contre la THA, Ministère de la Santé, Conakry, Guinée; 2 Centre Muraz, Ministère de la Santé, Bobo-Dioulasso, Burkina Faso; 3 Centre International de Recherche Développement sur l'Elevage en zone Subhumide, Bobo-Dioulasso, Burkina Faso; 4 Direction Nationale de l'Elevage, Ministère de l'Agriculture et de l'Elevage, Conakry, Guinée; 5 Institut de Recherche pour le Développement, UMR 177 IRD-CIRAD INTERTRYP, CIRDES Bobo-Dioulasso, Burkina Faso; 6 Institut de Recherche pour le Développement, UMR 177 IRD-CIRAD INTERTRYP, Campus International de Baillarguet, Montpellier, France; Makerere University, Uganda

## Abstract

Human African Trypanosomiasis (HAT) in West Africa is a lethal, neglected disease caused by *Trypanosoma brucei gambiense* transmitted by the tsetse *Glossina palpalis gambiensis*. Although the littoral part of Guinea with its typical mangrove habitat is the most prevalent area in West Africa, very few data are available on the epidemiology of the disease in such biotopes. As part of a HAT elimination project in Guinea, we carried a cross-sectional study of the distribution and abundance of people, livestock, tsetse and trypanosomes in the focus of Boffa. An exhaustive census of the human population was done, together with spatial mapping of the area. Entomological data were collected, a human medical survey was organized together with a survey in domestic animals. In total, 45 HAT cases were detected out of 14445 people who attended the survey, these latter representing 50.9% of the total population. Potential additional carriers of *T. b. gambiense* were also identified by the trypanolysis test (14 human subjects and two domestic animals). No trypanosome pathogenic to animals were found, neither in the 874 tsetse dissected nor in the 300 domestic animals sampled. High densities of tsetse were found in places frequented by humans, such as pirogue jetties, narrow mangrove channels and watering points. The prevalence of *T. b. gambiense* in humans, combined to low attendance of the population at risk to medical surveys, and to an additional proportion of human and animal carriers of *T. b. gambiense* who are not treated, highlights the limits of strategies targeting HAT patients only. In order to stop *T. b. gambiense* transmission, vector control should be added to the current strategy of case detection and treatment. Such an integrated strategy will combine medical surveillance to find and treat cases, and vector control activities to protect people from the infective bites of tsetse.

## Author Summary

Human African Trypanosomiasis (HAT) in West Africa is a lethal, neglected disease caused by *Trypanosoma brucei gambiense* transmitted by the tsetse fly *Glossina palpalis gambiensis*. Although the littoral part of Guinea with its typical mangrove habitat is the most prevalent area in West Africa, very few data are available on the epidemiology of the disease in such biotopes. We carried out a cross-sectional study of the distribution and abundance of people, livestock, tsetse and trypanosomes in the active focus of Boffa. We only found *T. b. gambiense* in the area, no other trypanosome. *T. b. gambiense* was found parasitologically in humans (45 cases), and suspected serologically in other humans and in two animals. Tsetse flies were present in high densities in places very frequented by humans, such as pirogue jetties, and watering points. Our results confirm the importance of medical surveys to find cases and treat them, but also point out the limit of strategies targeted at HAT patients only. If sleeping sickness is to be eliminated, a vector control component must be added to the strategy of case detection and treatment, and this latter must be directed to the population the most at risk.

## Introduction

Human African Trypanosomiasis (HAT, or sleeping sickness) is a lethal, neglected disease caused by a trypanosome (*Trypanosoma brucei gambiense* in West Africa) transmitted by an insect vector, the tsetse fly (*Diptera*: *Glossinidae*). In West Africa, Guinea is the country with the highest prevalence for HAT, especially in the littoral part [1], where the vector is *Glossina palpalis gambiensis*. From North to South, are found the active foci of Boffa, Dubreka and Forecariah, where infection rates in humans are generally around 0.5–1%, but can go up to 5% in some villages [2]–[4]. This littoral part is characterised by the mangrove habitat, with mangrove trees *Avicennia spp.*, *Rhizophora spp.* This mangrove habitat generally consists in both a Guinean savannah on the mainland, and of numerous islands harbouring tsetse flies, many of which are inhabited.

Very few data are available on the epidemiology of sleeping sickness in mangrove habitats, although this biotope is currently the one harbouring the highest prevalence in West Africa. There is also strong suspicion that HAT could also be highly prevalent in mangroves of Central Africa [5], [6]. There is growing information that the mangrove systems have proved very difficult to active detection and treatment programs. Hence, elimination, which represents actual WHO's aim, is challenged in such difficult habitats. However, it is not known so far if these difficulties are due to particular behaviour of the human communities living in mangrove which puts them at high contact with tsetse, or to particular tsetse populations or tsetse/trypanosomes interactions. Accordingly, as part of a major Government of Guinea/IRD programme to tackle HAT in the mangrove systems of West Africa with the support of WHO and the Bill and Melinda Gates Foundation, we carried a cross-sectional study of the distribution and abundance of people, livestock, tsetse and trypanosomes in the one of the important foci of HAT: the focus of Boffa.

## Materials and Methods

### Ethical statement

All the activities conducted in this study have been preceded by meetings of the National Control Program against HAT with authorities at the national, regional, and local level to explain the objective of the work and to obtain the agreement of the populations. Both the Ministry of health (for human survey) and the Ministry of Agriculture and Livestock, through the National Direction of Livestock have i.) approved our protocols, ii.) given their agreement and iii.) given the administrative authorizations for these activities. Then, every district and village was informed about the projects aims and activities by people from both ministries through various means including films, discussion forums, and slideshows.

All human samples were collected within the framework of medical surveys conducted by the national HAT control program (NCP) according to the national Guinean HAT diagnostic procedures of the Ministry of Health. No samples other than those collected for routine screening and diagnostic procedures were collected for the purposes of the present study. All human samples were anonymized. All participants were informed of the objective of the study in their own language. Hence the attendance of the population to the medical survey reflects the people who were there and who accepted to participate to the survey.

Only livestock owners who agreed to participate to the study had their animals sampled.

### Description of study area

In Boffa area, the geomorphology of the Rio Pongo estuary, with its extensive areas of sedimentation, has led to the development of halophile vegetation: the mangrove trees *Avicennia spp.*, *Rhizophora spp.* This mangrove habitat consists of numerous islands, many of which are inhabited, and which harbour *G. p. gambiensis* [7]. On these islands, people (mainly Soussou and Baga ethnic groups) cultivate irrigated rice, do salt extraction, cut wood, pick oysters, and fish. The mainland harbours a typical anthropised Guinean savanna landscape mainly colonized by *Elaeis Guineensis* and characterized by rivers bordered by gallery forests. Here, in addition to the above mentioned activities, people carry out market gardening in lower-lying areas and grow cassava, fonio, groundnut on the hillsides. Livestock does not constitute an important part of the economy, and looks more like a secondary activity. Mainly small ruminants (goats and sheep) are present, whereas cattle and pig breeding are restricted to some few families. Annual rainfall is around 3000 mm.

### Geographical survey

An exhaustive census of the human population was carried out. All the inhabitants of villages were counted and their houses geo-referenced using Global Positioning Systems (Garmin GPSmap76Cx, USA). In addition, all tracks, water sources (pumps, wells, holes of water, springs), bridges (car, pedestrian) and pirogue jetties (small, big, frequented by one family or several) were also mapped. Data on the number and types of domestic animals observed in each household was also recorded.

### Entomological survey

**Figure 1:**
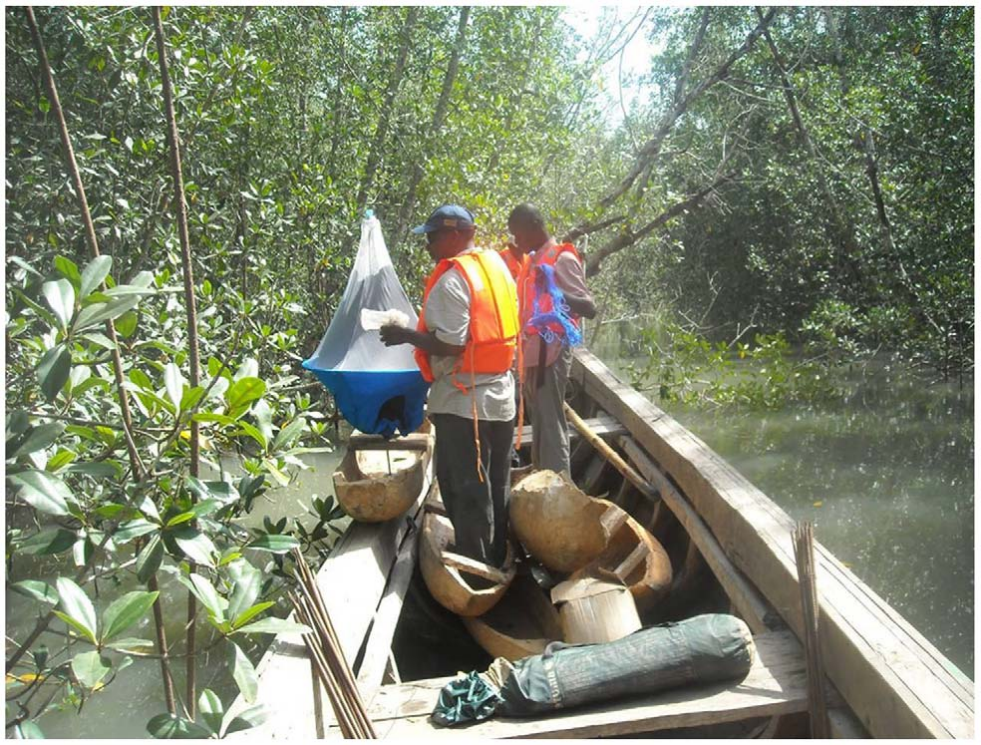
Setting up a floating trap in a mangrove channel.

The distribution and abundance of tsetse was assessed using biconical traps. In addition to the classical trap deployment made by walking on and between islands, the extensive network of channels meant that traps were also deployed by boat. So, in addition to the classical biconical traps, some floating traps [8] (see Fig. 1) were also deployed. A total of 344 traps were deployed for 48 h of consecutive trapping. When possible, cages were harvested daily. Tsetse were identified according to species and sex. To correct the great variability of the catches, data were normalized using a log+1 transformation. The median was then calculated with the 1^st^ and 3^rd^ quartiles, and were then back transformed. Females were individually aged by ovarian dissection, whereas the wing-fray method was used to estimate the male population's mean age. Males and females that were still alive were dissected in order to look for possible trypanosome infection using microscopy in the midgut, the proboscis, and the salivary glands.

### Population genetics of *G. palpalis gambiensis* in Boffa

As the Boffa area is constituted of a western and an eastern parts, separated by the Rio Pongo river which can reach more than one kilometer wide, the question was to know if the tsetse from the two parts were genetically differentiated, or alternatively if they belonged to one single panmictic population. Such information indeed has important consequences for designing tsetse control strategies (reviewed in [9]).

#### Samples and microsatellite markers

**Figure 2:**
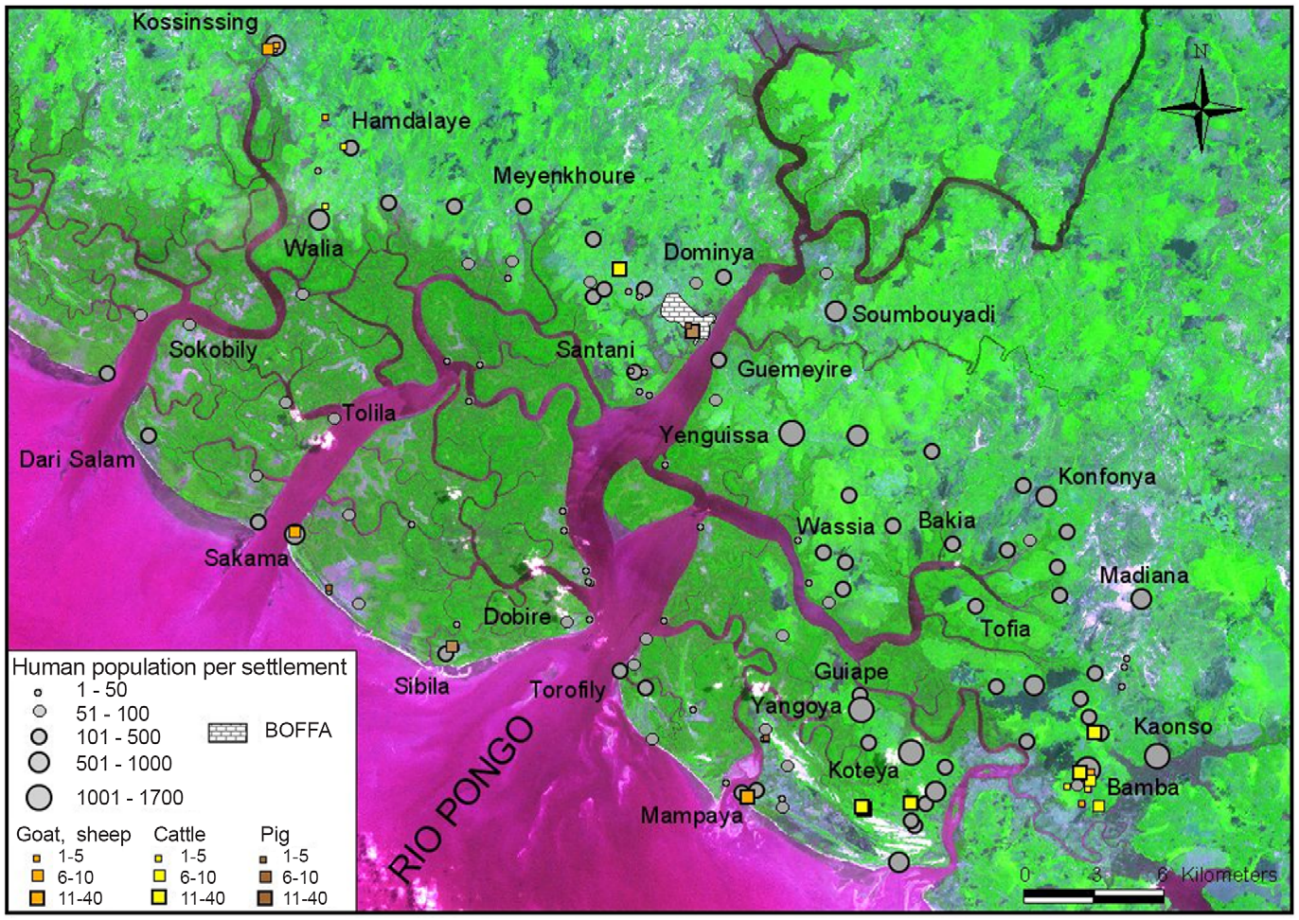
Human settlement and animal sampling in Boffa. The human settlement in Boffa is defined by 123 settlements comprising villages, hamlets and encampments harbouring variable population densities. Main places are located on the mainland (Yenguissa, Bamba, Kaonso) but also on some islands (Yangoya, Koteya). In general, islands are less populated than mainland, and the West bank of Rio Pongo (especially the islands part) shows a very low human settlement compare to the East bank. On the islands, there are a lot of small temporary (seasonal) settlements for salt extraction, fishing and rice cultivation. Most of the people on the mainland, who have activities on islands, use pirogue to go to the islands through mangrove channels. The location of the animals sampled in the area is also shown.

Tsetse flies were sampled in three sites that geographically cover the investigated area (Fig. 2): Dobire, South of the West bank (20 individuals: 11 females+9 males); Santani, North of the West bank (20 individuals: 17 females+3 males); and Yangoya South of the East bank (18 individuals: 10 females and 8 males).

Genetic analyses were performed using nine polymorphic microsatellite loci (for details see [10]): ×55.3 isolated from *G. p. gambiensis*, B3, XB104, XB110, C102 (kindly given by A. Robinson, IAEA), Xpgp13 and pgp24 from *G. p. palpalis*, GPCAG133 from *G. morsitans* and A10 kindly provided by G. Caccone. Thanks to a pedigree analysis from our laboratory-reared tsetse, 4 loci (55.3, B104, B110, pgp13, the name of which always begins with the letter “X”, were interpreted to be located on the X chromosome. Detailed procedures of genotyping can be found in [10].

#### Data analyses

The dataset was processed with Create V 1.1 [11] and converted into the appropriate format as needed. Wright's *F*-statistics [12] were estimated with Weir and Cockerham's unbiased estimators [13] under Fstat V 2.9.4 ([14], updated from [15]). *F*_IS_ is a measure of inbreeding of individuals relative to inbreeding of subsamples. It is therefore also a measure of deviation from panmixia and varies from −1 (all individuals are heterozygous for the same two alleles within each subsample) to +1 (all individuals are homozygous with at least two alleles in subsamples) and equals 0 when all subsamples conform to genotypic proportions expected under panmixia. *F*_ST_ measures inbreeding of subsamples relative to the total inbreeding. It results from the level of subdivision of the total population and is therefore also a measure of differentiation among subsamples. It varies between 0 (no differentiation) and 1 (all subsamples fixed for one or the other allele).

The significant departure from 0 of these parameter estimates was tested by randomisation procedures under Fstat with the *F*_IS_ based method and randomization of alleles between individuals within subsamples for *F*_IS_ and with *G* based test described in [16] and randomizing individuals between subsamplesfor testing differentiation.

Genotypic linkage disequilibrium (LD) between loci was tested through randomising each locus pair association. For each pair of loci the tests were combined across subsamples with the *G*-based procedure as recommended [17].

The X-linked loci were coded as missing data for *F*_IS_ and null allele analyses, and coded as homozygous for the allele present on the X for differentiation and LD tests.

Significant *F*_IS_ can be due to null alleles, stuttering or short allele dominance. We used MicroChecker V 2.2.3 [18] for stuttering and null allele detections. We tested how null alleles can explain the observed *F*_IS_ using estimates of null allele frequency following Brookfield's second method [19] as given by MicroCheker. We used these estimates to compute expected blank (non amplified null homozygotes) frequency assuming panmixia. For each locus, the sum of all expected blanks across subsamples was compared to the sum of all observed ones with an exact unilateral binomial test with the alternative hypothesis: there were not enough observed blank genotypes as compared to what would be expected under the hypothesis of null alleles in a panmictic population. This test was undertaken with R [20].

Confidence intervals (CI) were obtained using the standard error of estimates obtained by jackknife over subsamples (for each locus) or by bootstrap over loci (for the mean value), using Fstat, as described in [21].

Because differentiation between each of the three subsample pairs was what we wanted to test, we had to adjust each of the three differentiation tests with the sequential Benjamini and Hochberg correction [22] where the *k P*-values are ranked in increasing order and where the *i*th *P*-value *P_i_* is transformed into *P*′*_i_* = *k*×*P_i_*/*i*.

Effective population sizes were estimated following Waples and Do's method based on linkage disequilibrium and implemented in LDNe [23]. Other methods were not applicable.

### Human medical survey

HAT was diagnosed by mass screening of the population with the routinely used Card Agglutination Test for Trypanosomiasis (CATT, [24]) performed on capillary-collected blood (CATT-B). For each CATT-B positive person, additional blood was collected in heparinized tubes and a two-fold plasma dilution series in CATT buffer (CATT-P) was tested to assess the end titer, i.e. the highest dilution still positive. All individuals showing a CATT-P dilution end titer of 1/4 or greater (positive CATT-P), were submitted to microscopic examination of lymph node aspirate whenever swollen lymph nodes were present, and 350 µl of buffy coat was examined using the mini-anion exchange centrifugation test on buffy-coat (mAECT-BC, [25]). After centrifugation of the heparinized tube, one 1.5 ml eppendorf tube of plasma was sampled for every CATT-P positive subject for subsequent analyses (trypanolysis, see below). Staging of the disease was done by counting white cells in the cerebrospinal fluid: if the number of cells was <6, the patient was considered to be in the 1^st^ stage; early stage 2 was between 6 and 20 cells, and late stage 2 corresponded to cases with more than 20 cells.

At the end of the survey, all parasitologically positive subjects were treated according to the national treatment procedure taking into account staging results.

### Animal survey

During the initial geographical demographic survey, information had been gathered on the number and species of domestic animals present in the area. Power calculations suggested that a total sample size of 300 animals would provide a reliable estimate of the prevalence of trypanosome infection in the livestock population. For sampling, priority was given to good geographical coverage of the area, of course adapted to the low number of sites where domestic animals occurred (see fig. 2). Priority was then also given to sample animals known to harbour pathogenic trypanosomes, i.e. cattle and pigs, these latter having also been described as potential reservoir of human trypanosomes. For all animals sampled, blood was taken at the jugular vein in a 10 ml heparinized vacutainer tube. From this tube, a 75 µl capillary tube was taken, centrifuged. Packed cell volume (PCV) was measured, and parasitological diagnosis for trypanosomes was made according to the buffy-coat technique of Murray et al. [26], which has been recognized as one of the most sensitive. All the animals sampled have been given a free trypanocide treatment (Veriben at 3.5 mg/kg), except the pigs, for which this drug is not recommended. A deworming treatment (Bolumisol, Laprovet) was provided free to all animals sampled.

### Molecular characterization of the trypanosomes found in humans, animals, and tsetse

All the blood and plasma samples, together with tsetse samples collected in the focus of Boffa were first kept in a transportable cool box with ice, and then systematically transferred twice a day in a −20°C vehicle freezer (Engel). The samples were then transported back to CIRDES Bobo-Dioulasso where all the molecular analyses were done.

#### PCR in tsetse

From each dissected tsetse which had been found infected with trypanosomes using parasitological diagnosis, the salivary gland, proboscis, and midgut were put in individual eppendorf tubes containing sterile distilled water. On all these samples, DNA was extracted using two different protocols: the simple Chelex one (see [27] for details) and the DNeasy tissue kit (Qiagen) according to the instructions provided by the manufacturer.

DNA primers specific for *T. vivax* (TVW 1-2, [28]), *T. congolense* savannah (TCS 1-2, [29]), *T. congolense* West African riverine forest (TCF 1-2, [28]), *T. congolense godfrey*i (DGG1-2, [30]), *T. simiae* (TSM1-2, [28]), and *T. brucei sensu lato* (TBR 1-2, [29]) were used in single PCRs, as described in [27]. These diagnostic procedures are considered as very sensitive, since all these primers amplify repeated, satellite DNA sequences. The presence of *T. theileri* was also investigated using nested PCR based on unpublished ITS1 primers (Marc Desquesnes, unpublished data).

#### *T. b. gambiense* immune trypanolysis test on both human and animal plasma samples

Cloned populations of *T. b. gambiense* variable antigen type (VAT) LiTat 1.3 and *T.b. rhodesiense* VAT ETat 1.2R were used to test human and animal plasmas as previously described [3]. ETat 1.2R was a control for the absence of nonspecific trypanolytic activity of the tested plasma.

## Results

### Geographical survey

**Figure 3:**
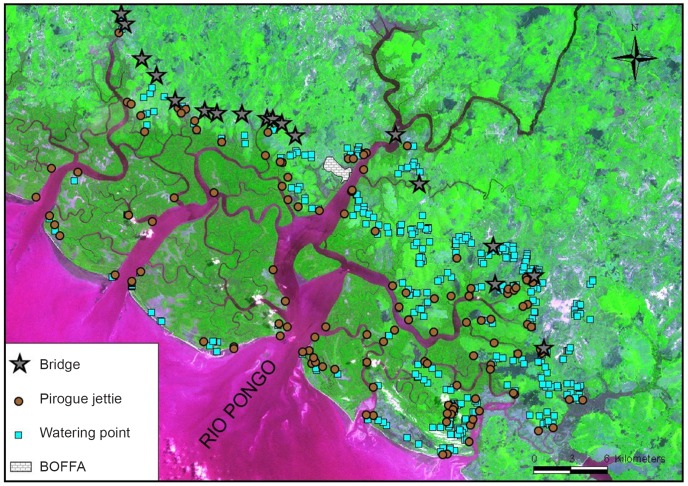
Geographic location of bridges, watering points and pirogue jetties in the focus of Boffa. All the bridges of the area are built on small rivers coming from the mainland and going towards mangrove ecosystem. The mangrove channels are the continuity of these little rivers. The high human mobility between mainland and islands is characterized by the 129 recorded and georeferenced pirogue jetties. The location of the 404 watering points (pumps, holes, springs, river points) are concentrated on the mainland with a very low number on the islands, especially on the West bank of Rio Pongo. All these points represent potential contact between humans and tsetse.

In total, 25 287 inhabitants (5 761 on the West side and 19 526 on the East side of the Rio Pongo) were censused. According to this census, in the Rio Pongo mouth (734 km^2^), the human density is around 34 inhabitants per square kilometer. Sixty seven percent of the population is located on the mainland, and 33% on the islands. Figure 2 shows high spatial heterogeneity in terms of human settlement. Most inhabitants from continental villages have a lot of activities in the mangrove (rice cultivation, salt extraction, fishing, etc.). Hence there is a very high human mobility between the mainland and the islands (for agricultural activities) and from the islands to the mainland (market, water supply etc…). This mobility, partly due to the fact that more than 50% of the population is under 25 years old, is illustrated by the important number of pirogue jetties, water point supplies, and bridges georeferenced (see Fig. 3), all these points being crucial for tsetse-human contact. Livestock production is not an important part of the local economy, and looks more like a secondary activity. Mainly small ruminants (goats and sheep) are present, whereas cattle and pig breeding are restricted to some few families.

### Entomological survey

#### Densities and infections

**Figure 4:**
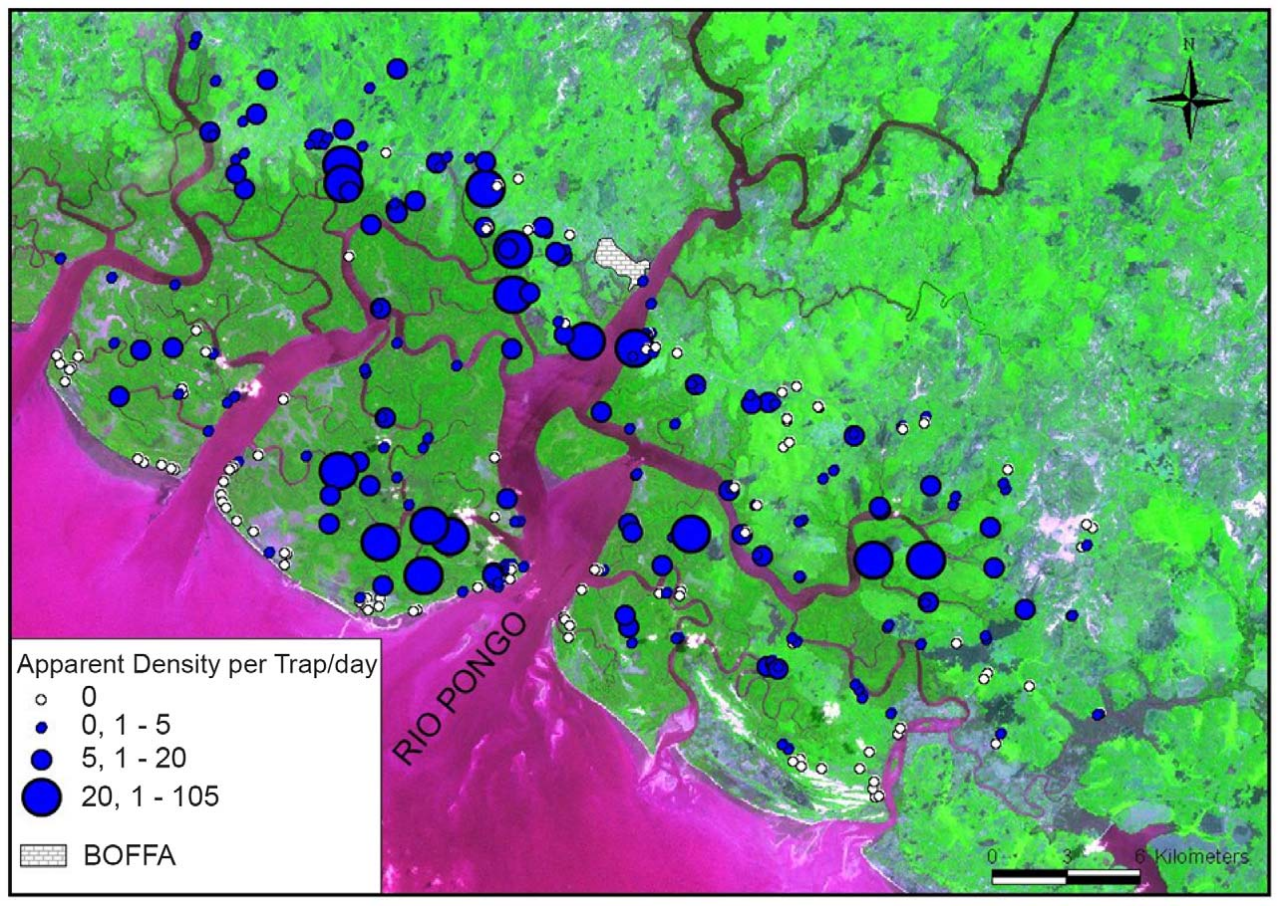
Location of traps and densities of tsetse caught during the entomological survey. On the mainland, tsetse (*Glossina palpalis gambiensis*) are found mainly along rivers and water points. In the mangrove, main densities are found on narrow mangrove channels and around frequented places such as pirogue jetties.

A total of 2730 flies was caught, consisting of 46% males (M) and 54% females (F), giving a F/M sex ratio of 1.17. All tsetse were *G. palpalis gambiensis*. These densities were very heterogeneous, ranging from 0 to 105 flies/trap/day (ftd), as can be seen in Fig. 4. The median of the catches was 1.5 with an interquartile range from 0 to 4.5 ftd. The greatest densities were generally observed either along small rivers inside villages or in savannah, or around natural water sources, wells, and jetties frequented by humans. In flooded mangrove areas, between the different islands, tsetse flies were also found at high densities inside the *Rhizophora* mangrove in the narrower parts of channels. As soon as the channels became wider, tsetse densities decreased.

A total of 874 tsetse were dissected (278 males and 596 females), thus representing 32% of the captured tsetse. Mean age of females was 35.2±17.37 days, whereas mean age of males was estimated to be 12 days. A total of 21 flies (8 males and 13 females) were found infected by trypanosomes (2.4±0.15%), among which 19 were infected in the midgut only, and 2 in both the midgut and proboscis (suggestive of a *Nannomonas* type trypanosome infection).

Out of these 21 infected tsetse, none of the infections could be identified as belonging to any of the pathogenic trypanosome tested by PCR (*T. brucei*, *T. congolense*, *T. simiae*, *T. godfreyi*, *T. vivax*), nor to the non-pathogenic *T. theileri*. This result was obtained using the two different protocols of DNA extraction described.

#### Population genetics of *G. palpalis gambiensis* in Boffa

None of the 36 LD tests was significant at the 5% level, meaning that the genotypic data at the 9 loci were not or weakly correlated.

There was a highly significant heterozygote deficit (*P*-value<0.001) that was reasonably explained by null alleles (all *P*-values>0.24).

None of the three differentiation tests per pair of subsample provided a significant *P*-value at the Benjamini and Hochberg level, and neither did the global differentiation test (all *P*-values>0.12), suggesting the different samples were not genetically differentiated, and thus belong to a unique, panmictic population at the scale of the focus.

The only reliable *N_e_* estimate was for Yangoya with an order of magnitude of 200 individuals, whatever the minimum allele frequency used (1, 2 or 5%). Pooling the three subsamples into a single one (since no genetic differentiation was found between samples), only alleles that have a frequency of 2% at least provided a reliable estimate with an order of magnitude of 1000 reproducing individuals.

### Human survey

In total, 14,445 persons were screened with CATT-B. Our population census estimated that 25287 people inhabit the area. In addition, 3088 people that were not included in the census attended the medical survey. Accordingly, we estimated that 50.9% (14445/28375) of the population was screened. A total of 324 subjects displayed a CATT-B positive result, among which 118 were CATT-P positive (mean seroprevalence = 0.82±0.08%). Among these 118 CATT-P positive subjects, 45 were confirmed to be HAT cases according to the parasitological tests (mean prevalence = 0.31±0.05%). The remaining 73 subjects (0.50%±0.06) were seropositive but parasitologically negative (hence labelled SERO), and did not receive treatment. Out of these 73 SERO, 14 (19.2%) were positive using the immune trypanolysis test (TL), hence labelled SEROTL+, indicating present or past contact with *T. b. gambiense*.

All the 45 HAT cases were TL positive (TL+), confirming a *T. b. gambiense* infection. Of these, 33 (73.3%) were positive for lymph node aspirate microscopic examination, and 45 (100%) were positive for mAECT-BC. Staging was performed on 43 HAT (two patients declined to come to Boffa hospital): 20 were diagnosed with late second stage of the disease, 9 were in early 2^nd^ stage, and 17 were in 1^st^ stage, with CSF white cell counts ranging from 0 to 1460/µl. Two late stage 2 twins who were 10 months old were treated with melarsoprol instead of NECT because of perfusion constraints. All other treated patients were given NECT. Neither important side effects nor death were registered during treatment of the patients.

**Figure 5:**
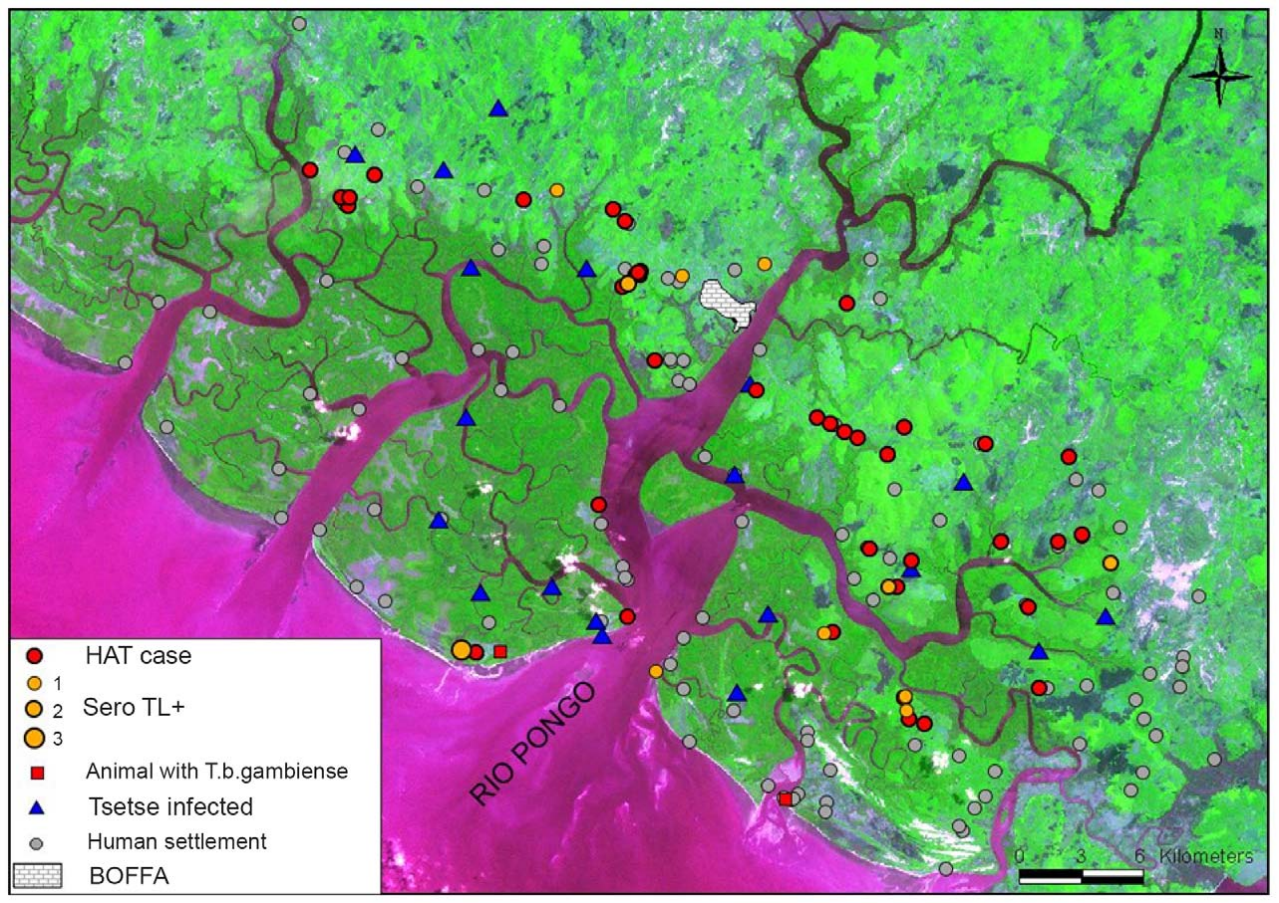
Spatial distribution of trypanosomes in humans, animals and tsetse. *T. b. gambiense* is found in HAT cases (here represented by the location of their house) and in both the seropositives-TL+ and two of the animals sampled. The trypanosomes observed parasitologically in tsetse could not be identified and are probably not *T. b. gambiense* (see text for details).

Figure 5 shows the spatial distribution of the HAT cases and of the SEROTL+. HAT patients and SEROTL+ subjects were not evenly distributed at the geographic scale of the focus, since most of them were located in the mangrove/mainland (savannah) interface. No significant differences were noted between the number of females (n = 20) and males (n = 25) with respect to HAT. Mean age of HAT cases was 28.7 (ranging from 10 months to 60 years old) with 31 HAT cases being 16 to 45 years old, and thus representing the active population. This means that 69% of the HAT cases were found in this active part of the population, although in this particular part of the population the attendance to the medical survey was low (40.29%).

### Animal survey

A total of 303 animals were sampled, consisting of 158 cattle (all being trypanotolerant Ndama breed), 49 pigs, 103 sheep and 3 goats, and were parasitologically examined for trypanosomes. Only one cattle was positive, showing a *T. theileri-*like infection. No pathogenic trypanosome was found. Mean PCV value of the 303 animals, all types confounded, was 33.3±6.75. Out of the 158 cattle sampled, only 4 had a PCV under 25, this value being the usual one under which a parasitological infection is suspected in areas where trypanosomiasis is found.

Out of 273 animal samples on which the trypanolysis test was implemented, two (one pig and one goat) were positive to LiTat 1.3, indicating a contact with *T. b. gambiense* (see Fig. 5).

## Discussion

In this study, we used parasitological, serological, and molecular tools, combined to geographical methods, to quantify the distribution and abundance of human and animal hosts, the vector, and the trypanosomes, in order to provide a comprehensive understanding of the epidemiological situation in the Guinean HAT focus of Boffa. The results show that the human population is young, very mobile, and that although *T. b. gambiense* is present and actively transmitted to humans in Boffa, it has not been found in tsetse, but has been suspected in two domestic animals, a pig and a goat. Remarkably, no pathogenic trypanosomes to animals have been observed in tsetse, nor in the 300 domestic animals sampled. Put together, and given the impossibility of eliminating *T. b. gambiense* by medical activities only, the results suggest that an integrated control strategy based on medical surveillance plus vector control will be required to protect people from HAT.

### Human settlement, landscape and HAT

The settlement and the landscape of Boffa focus are very similar to those which can be found on other parts of the Guinean littoral. From the Mellacoree river mouth in the south, at the border with Sierra Leone, to the Rio Compony river mouth in the north, near the border with Guinea Bissau, the Guinean littoral is characterized by a mangrove ecosystem, except for the headlands of Conakry and Cape Verga [31] and the important HAT foci in Guinea (Boffa, Dubréka and Forécariah) are all located in or around this mangrove ecosystem [2], [32]. Although the population of the littoral is multiethnic (Nalou, Baga, Temne ethnic groups), the Soussou group is clearly the most important. These populations conduct several activities in the mangrove that expose them to the bite of tsetse flies and so to the risk of getting HAT. In a recent study of Forecariah focus, in the southernmost part of the Guinean mangrove, the identified risk activities were rice cultivation, water supply at backwater and use of pirogue jetties [32]. In Boffa, high human mobility between the mainland and the islands is certainly an important factor that increases the risk of transmission from infected tsetse to humans, and that favors the spread of the disease. First, because this mobility is probably responsible for high human/tsetse contact at pirogue jetties and in narrow mangrove channels (highly human- frequented places with high tsetse densities), and second, because this mobility also contributes to a low attendance of population to medical surveys, in particular the active part of the population, which is also the most at risk. In many instances, when the medical survey team arrived in a village, where important sensitization always came along census campaign that preceded medical survey, many of the people were not present, simply because their activities in the mangrove were more important for them. It has to be noted, as part of the explanation, that these mangrove activities, have more constraints than classical agricultural ones on the mainland. For instance, people have to take into account tide hours as a first priority for their movements. These results can certainly be generalized to other HAT foci of the African coast with mangrove habitat, e.g. Equatorial Guinea, Gabon, etc.

### Tsetse distribution and population genetics

Considering tsetse distribution, two different types of habitats can be distinguished in the area: The northern and the southern ones. The northern one, on the mainland, looks like the classical distribution of the riverine species *G. palpalis gambiensis* in humid savannahs such as northern Ivory Coast, Burkina Faso, Mali, with tsetse being strongly associated to the forest galleries bordering water courses, and being almost absent from the savannah itself (e.g. [33], [34]). Here the highest densities are indeed found along the rivers, and near sources which offer conserved vegetation and suitable host abundance. On the opposite, in the southern part, when approaching the coast and mangrove channels, tsetse become progressively almost evenly distributed, with highest densities in narrow mangrove channels with *Rhizophora* vegetation, which represent hunting areas for tsetse, and also near natural watering points and pirogue jetties frequented by humans.

Given the absence of genetic differentiation observed between the tsetse sampled using 9 independent microsatellite loci, it is likely that Boffa provides a habitat for a single big panmictic tsetse population, which was *a priori* questionable given the wideness of Rio Pongo River (sometimes more than 1 km) which could have acted as a barrier between the two banks of the river. This confirms earlier observation on another sleeping sickness focus of the Guinean littoral, Dubreka, where mainland mangrove was also found to host a single large and panmictic tsetse fly population [10]. With such results, tsetse eradication at the scale of the focus of Boffa is clearly not the way to go because it is impossible to target the whole tsetse population, hence emphasis will be put to reduce (i.e. not eradicate) tsetse densities in order to reduce tsetse/human contact and stop *T. b. gambiense* transmission.

### Epidemiology of HAT in the focus of Boffa

The medical survey results confirmed the endemic situation in the Boffa focus with an overall prevalence of 0.3% in humans. Transmission of *T. b. gambiense* probably occurs mainly at the mangrove/savannah interface which is believed to be the main sites of human contact with tsetse flies. The active population (20–45 years old) is by far the most affected. This part of the population comprises 70% of the cases detected, although only 40% attended the medical survey. Transmission is probably linked to high human tsetse contacts occurring during human activities conducted in the mangrove, mainly around sites such as pirogue jetties, water supply points, and mangrove channels where many tsetse were captured. These sites will constitute priority targets for any vector control operation aiming at reducing tsetse/human contact.

The proportion of patients being in the 1st stage of the disease (39%) is greater than in other foci in littoral Guinea, since this proportion was only 2% in Dubreka [2], and 16% in Forecariah [4]. This suggests that in Boffa, HAT transmission is still active, and this underlines the pressing need for active intervention in this focus. Despite important efforts to sensitize the communities, only half of the whole population attended the medical survey and was therefore medically screened. This attendance even decreases to 40.29% when considering only the active population, which represents the majority of the cases (69%). Given that the overall prevalence was 0.3%, and assuming the prevalence is the same in the non screened population (which is very conservative), we therefore expect around 60 more HAT cases to be still living in the area. Moreover, application of TL proved that an additional 14 SERO-TL+ individuals (at least) indeed had a contact with *T.b. gambiense* and should be considered as potential additional carriers [35]. They confirm once more (see also [4], [36]) the potential impact SERO subjects can have in the maintenance of transmission in HAT foci, especially with HAT control strategies targeting HAT patients only. In the absence of any prophylactic treatment against sleeping sickness, this number of *T. b. gambiense* carriers is still growing since transmission is active. This emphasizes the need for intervention(s) that would overcome this constraint.

The presence of two 10 months old patients, the mother of whom was HAT diagnosed the year before (M.C., unpublished data), represents an additional indirect evidence for the anciently suspected existence of vertical *T. b. gambiense* transmission (reviewed in [37]).

### Characterization of trypanosomes in the different hosts

The absence of pathogenic trypanosomes in domestic animals was not expected, but is very likely given both their absence using parasitology (BCT method) on more than 300 animals sampled, and also given the high mean PCV values found. These results suggest that animal trypanosomiasis is not a major veterinary problem in this area. The same result had been found in Loos islands, near Conakry, where no domestic animal had been found infected with trypanosomes out of 104 sampled [38].

The fact that none of the infected tsetse had trypanosomes identified using PCR suggests that the trypanosomes circulating may come from reptiles for instance, such as *T. grayi* for which we did not do PCR. Reptiles (crocodiles, monitor lizards) are numerous in this mangrove area. But it may also suggest that unrecognised, possibly pathogenic, trypanosome species exist that were not identified. This can be particularly evoked for the two midgut+proboscis infections which would have been interpreted as a *Nannomonas* infection if only parasitological methods had been used.

Hence the only pathogenic trypanosome identified in Boffa focus is *T. b. gambiense*, its presence having been confirmed in humans, and in two domestic animals thanks to the trypanolysis test which is specific for *T. b. gambiense* [3]. *T. b. gambiense* has not been found in tsetse, confirming the usual (but poorly understood) very low (<0.1%) mature infection rates of *T. b. gambiense* in tsetse, even in active sleeping sickness foci ([39]. The same has been reported for *T. b. rhodesiense* (see [40]).

To summarize, our results suggest the absence of pathogenic trypanosomes in domestic animals in the focus of Boffa. We show the presence of *T. b. gambiense* in humans, and a contact between *T. b. gambiense* and some domestic animals (one pig and one goat in our study). The vectorial capacity of *G. p. gambiensis* in this focus seems very low, confirming what was found on another area of the Guinean littoral, the Loos islands, where Kagbadouno *et al.* [38] also reported an absence of trypanosome in the *G. p. gambiensis* dissected. This is in contrast with other areas where *G. p. gambiensis* occurs, such as the savannah areas of Burkina Faso or Mali for instance, where *G. p. gambiensis* can usually be found infected with animal trypanosomes, with infection rates generally ranging from 2 to 10% [41], [42]. The *G. p. gambiensis* from littoral Guinea may also be genetically different from the one from the West African savannah, this being currently investigated (P.S., unpublished data). Nonetheless, even a very small number of tsetse having a *T. b. gambiense* mature infection are able to spread these trypanosomes, since the infection by *T. b. gambiense* alters tsetse saliva and modifies the behaviour of the tsetse, favoring the risk of human infection [43]. The “good news” here is that, given the very low proportion of tsetse infected with *T. b. gambiense*, a human needs to be bitten a great number of times before being bitten by an infective tsetse. Hence, any intervention that will reduce tsetse numbers and tsetse/human contact will also reduce the number of tsetse infected, and will protect people from an infective bite.

Interventions directed against animal trypanosomiasis have been advocated as a useful entry point for controlling HAT [44]. However, in the littoral HAT foci of West Africa, and in many Central African foci, livestock are scant and/or trypanosomiasis is not an important constraint for livestock production. In these mangrove foci, interventions should be applied at a relatively small scale and have to be aimed specifically on eliminating HAT.

In conclusion, the prevalence of pathogenic trypanosomes in the human population, combined with low attendance at medical surveys and to an additional population of human carriers of *T. b. gambiense* living in the community, highlights the importance of implementing new strategies. We suggest that in order to stop *T. b. gambiense* transmission in Boffa and similar foci in West and Central Africa, vector control should be added to the current strategy of case detection and treatment. Such an integrated strategy of control will combine medical surveillance and vector control activities, the former finding and treating cases while the latter will protect people from the infective bites of tsetse. The recent development of insecticide-treated targets that are effective for *G. p. gambiensis* [45] offers the prospect of a method that can be applied in the mangrove systems. In addition there is an absolute need to follow up seropositive people, and to target more efficiently the population at risk if HAT is to be eliminated from the focus.
